# Osimertinib inhibits the MYLK4-mediated phosphorylation of CDKAL1 to suppress stemness and chemoresistance in rhabdomyosarcoma

**DOI:** 10.1038/s41392-025-02548-6

**Published:** 2026-01-22

**Authors:** Takuto Itano, Rongsheng Huang, Toshifumi Ozaki, Eiji Nakata, Atsushi Fujimura

**Affiliations:** 1 Department of Orthopaedic Surgery, Okayama University Graduate School of Medicine, Dentistry and Pharmaceutical Sciences, Okayama, Japan; 2 Department of Musculoskeletal Oncology, National Cancer Center Hospital, Chuo-ku, Tokyo Japan; 3 Department of Cellular Physiology, Okayama University Graduate School of Medicine, Dentistry and Pharmaceutical Sciences, Okayama, Japan; 4 Department of Trauma Orthopedics, The Second Hospital of Dalian Medical University, Dalian, Liaoning China; 5 Department of Molecular Physiology, Kagawa University Faculty of Medicine, Kagawa, Japan

**Keywords:** Sarcoma, Cancer stem cells, Paediatric cancer, Drug screening

**Dear Editor**,

Rhabdomyosarcoma (RMS) is a common pediatric soft-tissue sarcoma that often recurs or metastasizes despite aggressive multimodal therapy. Cancer stem cells (CSCs) are key contributors to these unfavorable outcomes. We previously demonstrated that CDKAL1 maintains CSC-like properties by facilitating eIF4F translation initiation complex assembly through its N-terminal domain, thereby enhancing SALL2 translation.^1^ Notably, the 5′ untranslated region (5′ UTR) of SALL2 mRNA contains a cytosine-enriched region that requires CDKAL1-dependent eIF4F complex assembly for efficient translation. Phosphorylation of the CDKAL1 N-terminus at threonine 43 (pT43) has been reported in multiple datasets, including PhosphoSitePlus, yet its role in either the canonical enzymatic activity of CDKAL1 (i.e., methylthiolation of tRNA^Lys^) or in cancer biology (i.e., CDKAL1-dependent translation of SALL2 mRNA) remains uncharacterized. Phosphoproteomic evidence suggests that MYLK4 may specifically phosphorylate this site in a sequence-dependent manner (PhosphoSitePlus).

Given this mechanistic uncertainty and the absence of pharmacological approaches to block this pathway, we hypothesized that targeting the MYLK4–CDKAL1 axis could modulate SALL2 translation and thereby suppress CSC-associated phenotypes in RMS. To test this hypothesis, we screened 2203 FDA-approved drugs via a dual-luciferase reporter containing the SALL2 5′ UTR upstream of the firefly luciferase gene, which enables monitoring of CDKAL1-dependent translation.^1^ Among the top candidates, we prioritized osimertinib, a third-generation epidermal growth factor receptor (EGFR) inhibitor, because of reported links between EGFR, MYLK4, and drug resistance.^2,3^ Immunoblotting and reverse transcription‒quantitative polymerase chain reaction (RT‒qPCR) confirmed that osimertinib suppressed SALL2 expression at the translational level,^4^ reduced sphere formation,^4^ and inhibited eIF4F assembly in RD cells (Fig. 1a).

**Fig. 1:**
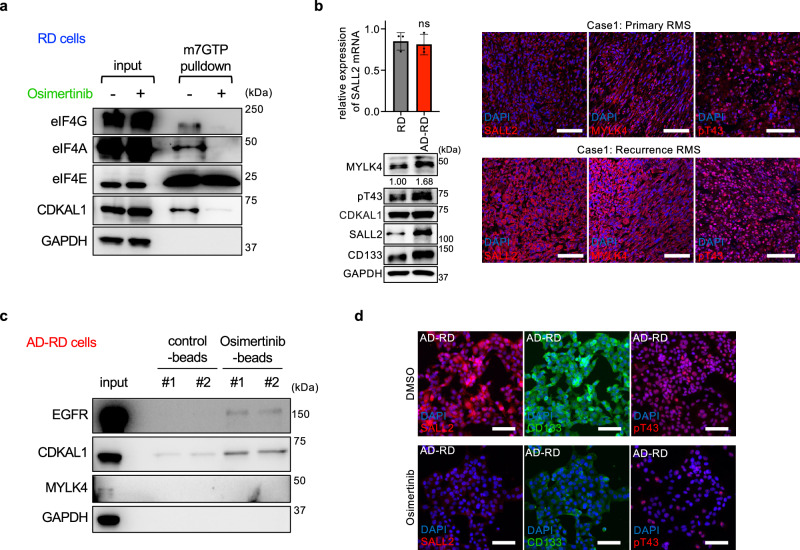
Identification of osimertinib as an inhibitor of the MYLK4–CDKAL1–SALL2 axis. **a** Immunoblotting of m^7^GTP precipitates from osimertinib-treated RD cells (2.5 µM, 24 h) indicated that osimertinib inhibited translation initiation complex formation. **b** SALL2 mRNA and protein expression levels of MYLK4, pT43-CDKAL1 (pT43), CDKAL1, SALL2, and CD133 in RD and AD-RD cells, as determined by RT‒qPCR (mean ± SD, *n* = 3) and immunoblotting, respectively. GAPDH served as a loading control. Statistical significance: ns, *p* ≥ 0.05. Fluorescent immunostaining of SALL2, MYLK4, and pT43-CDKAL1 in paired primary and recurrent rhabdomyosarcoma samples from the same patient. **c** Pull-down assay using osimertinib-conjugated or control beads incubated with lysates from AD-RD cells, followed by immunoblotting for CDKAL1, MYLK4, EGFR, and GAPDH (negative control). **d** Representative immunofluorescence images of AD-RD cells treated with 1 µM osimertinib for 48 h and stained with antibodies against CD133, SALL2, and pT43-CDKAL1. Scale bars: 100 μm

RMS CSCs are known to exhibit chemoresistance.^5^ To examine whether the MYLK4–CDKAL1–SALL2 pathway is involved in CSC-related traits, we established an actinomycin D-resistant RD cell line (AD-RD) by gradually increasing the drug concentration.^4^ Compared with parental RD cells, AD-RD cells presented elevated CD133 and SALL2 expression (Fig. 1b) and greater self-renewal capacity,^4^ supporting earlier findings linking drug resistance to CSC phenotypes.^5^ While total CDKAL1 expression was not notably increased, pT43-CDKAL1 levels were strongly elevated in AD-RD cells, as was MYLK4 expression (Fig. 1b). SALL2 expression was also upregulated in a translation-dependent manner, indicating the activation of the MYLK4–CDKAL1–SALL2 pathway in resistant cells (Fig. 1b). To further examine whether activation of the MYLK4–CDKAL1–SALL2 axis also occurs in clinical samples, we performed fluorescent immunostaining for SALL2, MYLK4, and pT43-CDKAL1 in paired tumor samples obtained from the same patient at initial diagnosis and at recurrence. As shown in Fig. 1b, the recurrent tumors presented markedly stronger fluorescence signals for all three proteins than did the primary tumors. These findings indicate that recurrent rhabdomyosarcoma lesions following standard therapy exhibit enhanced activation of the MYLK4–CDKAL1–SALL2 pathway, which is consistent with the mechanism observed in our resistant cell model.

To determine whether osimertinib directly inhibits MYLK4-mediated CDKAL1 phosphorylation, we conducted an in vitro kinase assay using recombinant proteins. Purified CDKAL1 and MYLK4 were incubated with the top-ranked compounds and first-generation EGFR-tyrosine kinase inhibitors tested in our screen, and pT43-CDKAL1 levels were assessed by immunoblotting. Because the assay contained only purified CDKAL1 and MYLK4 proteins and not EGFR, the reaction was independent of EGFR activity. Osimertinib emerged as the most effective inhibitor of pT43-CDKAL1.^4^ Coimmunoprecipitation of Myc-CDKAL1 and FLAG-MYLK4 in RD cell lysates revealed that osimertinib reduced their interaction, indicating direct inhibition of the protein–protein interaction, independent of EGFR inhibition.^4^ To further explore how osimertinib inhibits MYLK4-mediated CDKAL1 phosphorylation, we synthesized an osimertinib–COOH conjugate and immobilized it on NH_2_-activated beads. Lysates from AD-RD cells were incubated with either control (acetyl-mask) or osimertinib-conjugated beads, followed by immunoblotting of bound proteins. As shown in Fig. 1c, osimertinib bound directly to CDKAL1 but not to MYLK4. These results indicate that osimertinib inhibits the MYLK4–CDKAL1 interaction by directly binding to CDKAL1 and likely blocking its MYLK4-binding interface.

Compared with parental RD cells, AD-RD cells exhibited significantly greater sensitivity to osimertinib, with reduced sphere-forming capacity observed even at lower drug concentrations.^4^ Osimertinib also inhibited eIF4F assembly in AD-RD cells,^4^ suggesting that resistant cells rely more heavily on MYLK4-dependent pT43-CDKAL1 to maintain CSC traits. Consistent with these findings, knockdown of MYLK4 in AD-RD cells led to a marked reduction in pT43-CDKAL1 and SALL2 protein levels, accompanied by decreased self-renewal under anchorage-independent conditions.^4^ We next evaluated the synergistic effects of osimertinib with a frontline chemotherapeutic agent in AD-RD cells. Actinomycin D, a transcription inhibitor, reduced CDKAL1 expression but had no effect on pT43-CDKAL1.^4^ In contrast, combination treatment with osimertinib and actinomycin D markedly decreased pT43-CDKAL1 levels and cell proliferation.^4^ Immunofluorescence analyses of osimertinib-treated AD-RD cells revealed reduced expression of SALL2, CD133, and pT43-CDKAL1 (Fig. 1d). These results suggest that osimertinib may exhibit a more potent anticancer effect in RMS with high CSC potential and a tendency for recurrence, considering its ability to inhibit CDKAL1 Thr43 phosphorylation.

In summary, osimertinib disrupts the MYLK4–CDKAL1 interaction, inhibits pT43-CDKAL1 phosphorylation, and suppresses CDKAL1-dependent SALL2 translation, thereby impairing CSC maintenance and enhancing chemosensitivity in RMS. Given its FDA approval and well-established safety profile, osimertinib holds promise for rapid repurposing as a therapeutic strategy for chemoresistant RMS.

## Supplementary information

Supplementary Materials for Osimertinib inhibits the MYLK4-mediated phosphorylation of CDKAL1 to suppress stemness and chemoresistance in rhabdomyosarcoma

## Data Availability

All additional figures that could not be included in the main figure due to the Letter format requirements have been deposited in Figshare and publicly available at the following 10.6084/m9.figshare.30741332.
