# Supplementary material for: Osimertinib inhibits the MYLK4-mediated phosphorylation of CDKAL1 to suppress stemness and chemoresistance in rhabdomyosarcoma
Source: Signal Transduct Target Ther. 2026 Jan 22;11:26. doi: 10.1038/s41392-025-02548-6 (PMC12824139; doi:10.1038/s41392-025-02548-6)
Supplement: Supplementary file 1 — Supplementary Materials for Osimertinib inhibits the MYLK4-mediated phosphorylation of CDKAL1 to suppress stemness and chemoresistance in rhabdomyosarcoma [file 41392_2025_2548_MOESM1_ESM.docx]

**Supplementary Materials for**

**Osimertinib inhibits the MYLK4-mediated phosphorylation of CDKAL1 to suppress stemness and chemoresistance in rhabdomyosarcoma**

Takuto Itano, Rongsheng Huang, Toshifumi Ozaki, Eiji Nakata, Atsushi Fujimura*

*Correspondence: fujimura.atsushi@kagawa-u.ac.jp

**This file includes:** Materials and methods, References

**Materials and Methods:**

**Cell culture and reagents**

Human RMS cell line RD was obtained from the Japanese Collection of Research Bioresources (JCRB). 293FT cells were purchased from Invitrogen (R70007) for lentivirus production and Expi293 cells were purchased from Thermo Fisher Scientific (A14635) for recombinant protein preparation^1^. RD and AD-RD cells were cultured at 37 °C containing 5% CO_2_ in high-glucose Dulbecco’s modified Eagle’s medium (Fujifilm-Wako, 041-30081) supplemented with 10% fetal bovine serum (Nichirei Biosciences, 175012) and 1% penicillin/streptomycin/L-glutamine (Fujifilm-Wako, 161-23201). Expi293 cells were cultured at a 37 °C incubator with ≥ 80% relative humidity and 8% CO_2_ in Expi293 Expression Medium (Thermo Fisher Scientific, A1435101) on an orbital shaker^1^. Osimertinib (S7297), Ozanimod (S7952), Perhexiline (S6959), Lomitapide (S7635), Gefetinib (S1025), and Erlotinib (S7786) were purchased from Selleck^1^. Actinomycin D (018-21264) was purchased from Fujifilm-Wako and Sigma-Aldrich^1^.

**Immunoblotting**

Immunoblotting analyses were performed as previously described^2^ with minor modifications. Briefly, Cells were lysed in Triton X-100–based lysis buffer supplemented with protease and phosphatase inhibitors (Roche). After clarification by centrifugation, lysates were mixed with SDS sample buffer, boiled, and subjected to SDS-PAGE followed by transfer to PVDF membranes (Bio-Rad). Membranes were blocked with 0.5% BSA or milk in TBST, incubated with primary antibodies overnight at 4°C, washed, and then incubated with HRP-conjugated secondary antibodies. Signals were detected using ECL reagents (Bio-Rad) and visualized with ChemiDoc (Bio-Rad). The following are the antibodies used in immunoblotting analyses, listed as [Protein/Source/Identifier]: [GAPDH/Proteintech/60004-1-Ig], [CD133/Proteintech/18470-1-AP],[CD133/Proteintech/66666-1-Ig], [CDKAL1/Proteintech/22988-1-AP], [pT43-CDKAL1/House made/(-)], [MYLK4/Proteintech/24309-1-AP], [Myc tag/Medical&Biological Laboratories/M192-3], [DYKDDDDKtag/Fujifilm-Wako/012-22384], [SALL2/Bethyl Laboratories/A303-208A], [eIF4A1/Cell Signaling Technology/2490S], [eIF4G1/ Cell Signaling Technology/2469S], [eIF4E/Cell Signaling Technology/2067S], [Anti-Rabbit IgG, HRP-linked/Cell Signaling Technology/7074P2], [Anti-Mouse IgG, HRP-linked/Sigma-Aldrich/A9044]^1^. pT43-CDKAL1 was produced by injecting an antigen peptide [KVRRRN(pT)QKYLQE] into a rabbit and was purified using an antigen-affinity column (Scrum Inc.).

**Quantitative real-time PCR (qPCR) for mRNA expression analysis**

Total RNA was extracted using TRIzol (Invitrogen) and treated with DNase I. cDNA was synthesized using PrimeScript RT Master Mix (Takara), and qRT-PCR was performed with Luna Universal qPCR Master Mix (NEB). Gene expression was quantified on a Rotor-Gene system (Qiagen) and normalized to 18S rRNA. The oligonucleotide sequences used in this study were [SALL2: CCCCTGATCTTGGAAGAGCTA; CACCGTCTGGCCTAAGGAG], and [18S rRNA: GTAACCCGTTGAACCCCATT; CCATCCAATCGGTAGTAGCG].

**Immunostaining**

Immunostaining was performed as previously described^3^ with minor modifications. Briefly, cells grown on chamber slides or deparaffinized tissue sections were fixed, permeabilized, blocked, and incubated with primary antibodies overnight at 4 °C, followed by fluorescent secondary antibodies and DAPI counterstaining. Images were acquired using a Zeiss LSM780 confocal microscope and analyzed with ZEN software. The following are the antibodies used for immunostaining, listed as [Protein/Source/Identifier]: [CD133/Proteintech/66666-1-Ig], [pT43-CDKAL1/House made/(-)], [SALL2/Bethyl Laboratories/A303-208A], [MYLK4/Proteintech/24309-1-AP], [Donkey anti-Mouse IgG, Alexa Fluor 488/Thermo Fisher Scientific/A32766], [Donkey anti-Rabbit IgG, Alexa Fluor Plus 594/Thermo Fisher Scientific/A32754].

**M7GTP pulldown assay**

eIF4E and associated proteins were pulled down using m7GTP-agarose beads (Jena Bioscience). Cells were lysed in Triton-based buffer with protease/phosphatase inhibitors, clarified by centrifugation, and incubated with m7GTP beads at 4 °C. After washing, bound proteins were eluted with SDS sample buffer and analyzed by immunoblotting.

**Colony and sphere formation assay^1^**

For colony formation, 5,000 cells were seeded in 6-well plates and cultured for several days, then fixed, crystal-violet stained, and quantified using ImageJ.
For sphere formation, 1,000 cells were plated in ultra-low attachment plates (Corning) in serum-free stem cell medium and cultured for 7 days; spheres >100 µm were counted.

**Recombinant protein preparation and in vitro kinase assay^1^**

FLAG-tagged MYLK4 was expressed in Expi293 cells and purified using anti-FLAG affinity gel (Merck). Myc-tagged CDKAL1 was expressed in RD cells and isolated using anti-Myc magnetic beads (MBL). For kinase assays, bead-bound CDKAL1 was incubated with purified MYLK4 in kinase buffer containing ATP, with or without drug treatment. Reactions were performed at 37 °C and terminated by boiling in SDS sample buffer, followed by immunoblot analysis.

**Preparation of Osimertinib-conjugated beads**
Osimertinib–COOH was synthesized by Kishida Chemical Co., Ltd. (Osaka, Japan). NH_2_-activated beads were purchased from Tamagawa Seiki Co., Ltd. (Nagano, Japan). Conjugation of osimertinib–COOH to NH₂ beads was performed according to the manufacturer’s protocol. Briefly, osimertinib–COOH was activated with *N*-hydroxysuccinimide (HOSu) and 1-ethyl-3-(3-dimethylaminopropyl)carbodiimide (EDC), followed by coupling to NH_2_ beads in the presence of acetic anhydride.

**Clinical specimens**

Tumor and peritumor tissues were obtained from patients with RMS treated at Okayama University Hospital. All procedures adhered to the ethical guidelines approved by the Ethical Committee of the Okayama University Graduate School of Medicine, Dentistry and Pharmaceutical Sciences and Okayama University Hospital (Approval numbers: K1509-037, K2304-022). Informed consent was obtained from all participants or their legal guardians.

**References**

1. Itano, T. et al. supplementary figure: Osimertinib inhibits the MYLK4-mediated phosphorylation of CDKAL1 to suppress stemness and chemoresistance in rhabdomyosarcoma. *Figshare*, https://doi.org/10.6084/m9.figshare.30741332.
2. Fujimura, A. *et al.* In Vitro Studies to Define the Cell-Surface and Intracellular Targets of Polyarginine-Conjugated Sodium Borocaptate as a Potential Delivery Agent for Boron Neutron Capture Therapy. *Cells* **9** (2020).
3. Yamamoto, T. *et al.* 2-Methylthio Conversion of N6-Isopentenyladenosine in Mitochondrial tRNAs by CDK5RAP1 Promotes the Maintenance of Glioma-Initiating Cells. *iScience* **21**, 42-56 (2019).
